# Knowledge, attitudes, and practices regarding osteoporosis among hormone receptor-positive breast cancer patients: a cross-sectional study

**DOI:** 10.3389/fpubh.2026.1741278

**Published:** 2026-03-05

**Authors:** Xiaoyan Chen, Rongfang Xu, Xiangyang Sun, Xianghua Huang, Lingli Chen, Jibing Liu, Xinghui Li, Lulu Pan, Ying Cheng

**Affiliations:** 1 Department of Breast Surgery, Nantong Tumor Hospital, Affiliated Tumor Hospital of Nantong University, Nantong, China; 2 Nursing Department, Nantong Tumor Hospital, Affiliated Tumor Hospital of Nantong University, Nantong, China; 3 Office of the President of Nantong Tumor Hospital, Affiliated Tumor Hospital of Nantong University, Nantong, China; 4 Department of Scientific Research and Teaching Management, Nantong Tumor Hospital, Affiliated Tumor Hospital of Nantong University, Nantong, China

**Keywords:** attitudes, hormone receptor-positive breast cancer, knowledge, osteoporosis, patient, practices

## Abstract

**Objective:**

To investigate the knowledge, attitudes, and practices (KAP) concerning osteoporosis among hormone receptor-positive (HR+) breast cancer patients.

**Methods:**

A cross-sectional study was conducted from November, 2023, to March, 2024 among HR+ breast cancer patients to assess their KAP regarding osteoporosis. Structural equation modeling (SEM) was employed to assess the relationships among the KAP dimensions.

**Results:**

A total of 758 HR+ breast cancer patients were invited, 610 completed the questionnaire (response/completion rate: 80.5%), and 491 questionnaires were retained for the final analysis after prespecified data cleaning (analytic inclusion rate: 64.8%). HR+ breast cancer patients had insufficient knowledge (mean 9.69, SD 5.93), moderate attitudes (mean 29.50, SD 4.55), and moderate practices (mean 35.85, SD 4.75). SEM showed that knowledge directly influenced attitudes (β = 0.509, *P* < 0.001), while both knowledge (β = 0.585, *P* < 0.001) and attitudes (β = 0.402, *P* < 0.001) had direct effects on practices.

**Conclusions:**

HR+ breast cancer patients exhibit insufficient knowledge, positive attitudes, and suboptimal practices regarding osteoporosis.

## Introduction

Breast cancer remains a major global health challenge, ranking among the leading causes of cancer-related deaths in women. In 2022, it accounted for 2.3 million new cases and 670,000 deaths worldwide (1). Although the incidence in China is lower than in Western countries, Chinese women still represent 12.2% of global new cases, with an increasing trend among younger individuals that contributes substantially to premature mortality and societal burden (2).

Breast cancer is a heterogeneous disease, broadly classified as hormone receptor-positive (HR+), HER2-positive, or triple-negative (3), with HR+ subtypes comprising approximately 75%−80% of all cases (4, 5). Endocrine therapy, the standard treatment for HR+ breast cancer, includes selective estrogen receptor modulators such as tamoxifen and aromatase inhibitors. Estrogens play a key role in maintaining bone homeostasis by suppressing bone resorption and supporting bone formation, and reduced estrogen signaling can accelerate bone loss. In addition to aromatase inhibitors, selective estrogen receptor degraders (SERDs) further inhibit estrogen receptor signaling and may adversely affect bone health. Tamoxifen exerts bone-protective effects in postmenopausal women but may induce bone loss in premenopausal women (6) whereas aromatase inhibitors reduce estrogen synthesis and cause annual bone losses of 2.2%−2.6% in the lumbar spine and 1.7%−2.1% in the hips (7). Consequently, cancer treatment-induced bone loss (CTIBL) has become one of the most common long-term complications among breast cancer survivors, contributing to fragility fractures, hospitalization, disability, and increased mortality risk (8, 9). In current clinical practice, bone health monitoring is recommended for patients receiving endocrine therapy, including baseline and follow-up bone mineral density assessment (DXA) and evaluation of vitamin D status when appropriate. Preventive or therapeutic strategies typically include patient education, adequate calcium and vitamin D intake, weight-bearing exercise and fall prevention, and pharmacologic interventions such as antiresorptive therapy for patients at elevated fracture risk.

The Knowledge, Attitudes, and Practices (KAP) model suggests that individual behaviors are influenced by one's knowledge and attitudes, which in turn dictate their practices. This framework is particularly valuable in public health for understanding health-related behaviors through KAP surveys, which assess both knowledge and risk perception. This approach is essential for elucidating the complex behaviors associated with health management and promotion (10–12). HR+ breast cancer patients undergoing endocrine therapy face increased risks of bone density loss and fractures due to the unwanted side effects related to treatment. These medications effectively inhibit cancer cell growth but can lead to significant osteoporosis. Additionally, menopausal bone loss further exacerbates the reduction in bone density. Studying the KAP of this demographic can identify knowledge gaps and misconceptions, enabling healthcare professionals to tailor health education and clinical interventions more effectively. Such measures are crucial not only for enhancing disease management efficiency but also for improving patients' quality of life. By reducing the complications associated with osteoporosis, these interventions aim to improve overall treatment outcomes and patient satisfaction. Recently, Ma et al. (13) conducted a study on knowledge, attitudes, and practices regarding osteoporosis, providing valuable insights into this area. However, our study specifically targets HR+ breast cancer patients, a more focused population, and examines a geographically different cohort in Nantong City, China.

Despite the importance of these therapeutic goals, there is a notable lack of KAP studies focused on osteoporosis prevention and reduction among HR+ breast cancer patients. Therefore, this study aimed to investigate the KAP concerning osteoporosis within this group, addressing the need for targeted research that can lead to more effective health outcomes.

## Materials and methods

### Study design and participants

The current cross-sectional study was conducted from November, 2023, to March, 2024 at Nantong Tumor Hospital in Nantong City, China, focusing on patients receiving endocrine therapy for hormone receptor-positive breast cancer in the maintenance phase, defined as having completed surgery and acute chemotherapy/radiotherapy and continued endocrine therapy for at least 3 months, including both adjuvant therapy and advanced maintenance therapy. During the study period, potentially eligible patients were screened based on clinical records and physician confirmation, and those meeting the inclusion criteria were approached and invited to participate on a voluntary basis. Ethical approval (Approval No: TONGLUNSHENSHI [Keyan] 2022-065) was obtained from the Ethics Committee of the Nantong tumor Hospital, and informed consent was secured from all subjects.

Inclusion criteria encompassed patients with confirmed hormone receptor-positive breast cancer (stage I–IV) who were receiving endocrine therapy in the long-term maintenance phase after completion of initial treatment (e.g., surgery, chemotherapy and/or radiotherapy), individuals with at least primary school education, and patients possessing normal mental capacity, adequate communication skills, and understanding of their medical condition. Only female patients were eligible; male breast cancer patients were excluded to ensure cohort homogeneity, given their low prevalence and different physiological response to endocrine therapy. Because endocrine therapy is also a standard treatment option for some patients with advanced HR+ disease, patients with stable metastatic disease receiving endocrine therapy were eligible for inclusion. A prior diagnosis of osteoporosis was not a reason for exclusion to reflect the real-world clinic population, and osteoporosis status was collected in the baseline questionnaire. Osteoporosis status was self-reported (yes/no/uncertain) and was not systematically confirmed by mandatory DXA testing or by medical record adjudication for all participants in this study. Additionally, voluntary participation was a requisite for inclusion. Exclusion criteria comprised patients with an ECOG performance status > 3 or those receiving end-of-life palliative care who are unable to complete the survey, patients with diagnosed cognitive impairment, dementia, or severe psychiatric disorders (e.g., schizophrenia) that prevent informed consent, patients diagnosed with concurrent malignancies other than breast cancer, and those afflicted with severe degenerative bone or joint disorders.

### Procedures

The questionnaire underwent multiple revisions, informed by feedback from eight experts in breast cancer treatment. This panel included four medical experts—two internal medicine specialists, one surgeon, and one radiotherapy specialist—and four nursing experts—two nursing management specialists and two clinical nursing specialists. Before the formal survey, a pilot study was conducted with a random sample of 30 patients diagnosed with Hormone Receptor-Positive Breast Cancer Patients. The questionnaire was pre-tested and refined based on results from the pilot study, achieving a reliability coefficient of 0.841.

The final questionnaire, in Chinese, comprised four dimensions. Firstly, participants' demographic information was collected through nine items. Secondly, the knowledge dimension included 13 questions, where correct responses to single-choice questions were awarded 1 point and incorrect or unclear responses received 0 points. Additionally, questions 1, 7, 8, and 11 were multiple-choice items; each correct option was awarded 1 point. Across these four items, there were 16 correct options in total. Therefore, the maximum knowledge score was 25 points (9 single-choice items × 1 point + 16 correct options across multiple-choice items). Thirdly, the attitudes dimension consisted of nine items, with the first item being open-ended and the remainder utilizing a five-point Likert scale to assess attitudes, yielding scores between 8 and 40 points. Lastly, the practices dimension comprised 10 items covering diet, exercise, and sun exposure, with the first item offering a binary choice (*A* = 1; *B*/*C* = 0) and items 2–10 rated on a five-point Likert scale (1–5), leading to scores ranging from 9 to 46 points.

### Score categorization for interpretation

To provide a standardized basis for qualitative descriptions, we categorized KAP scores using the modified Bloom's cut-off points based on the percentage of the maximum possible score: poor/insufficient (< 60%), moderate (60%−79%), and good/positive (≥80%). Accordingly, for the knowledge scale (0–25), scores < 15 were classified as insufficient, 15–19 as moderate, and ≥20 as good. For the attitudes scale (8–40), scores < 24 were classified as poor, 24–31 as moderate, and ≥32 as good. For the practices scale (9–46), scores < 28 were classified as poor, 28–36 as moderate, and ≥37 as good. These categories were used only to support the narrative qualitative labels, while continuous scores were retained for statistical analyses.

During routine outpatient visits, including waiting for consultation or follow-up appointments for endocrine therapy, eligible patients were approached by trained investigators and invited to participate. The questionnaire was implemented on a digital platform (Questionnaire Star) and accessed via a QR code after informed consent was obtained. Following submission, investigators checked the questionnaire only for completeness (e.g., missing items) and obvious logical inconsistencies. No guidance, prompting, or feedback on item content was provided during completion, and investigators did not intervene in or influence participants' responses. To enhance data accuracy, entries were double-checked during data entry to minimize potential biases introduced by personnel. To ensure patient confidentiality and informed consent protection, information that directly identified patients (such as names and ID numbers) was separated from the study data immediately after collection. Informed consent was presented on the first page of the questionnaire, and only participants who provided informed consent proceeded to the formal questionnaire content; otherwise, they were automatically redirected out of the survey. We defined the response (completion) rate as the proportion of completed questionnaires among all invited participants, and we reported the analytic inclusion rate as the proportion of invited participants retained after prespecified data cleaning.

### Data quality control and handling of contradictory responses

To ensure reproducible scoring, we applied pre-specified data-cleaning rules before analysis. Participants were excluded if they were not hormone receptor-positive breast cancer patients based on screening/clinical verification. In addition, for multiple-choice knowledge items, responses were considered logically contradictory if the respondent selected “Uncertain” together with one or more specific answer options within the same item (e.g., in item K8, selecting option “Uncertain” and simultaneously selecting other options). Such responses could not be scored unambiguously under the predefined grading scheme; therefore, these questionnaires were excluded from the analytic dataset.

### Sample size determination

The required sample size was estimated using the formula:


$$\begin{array}{l} n=\frac{z^{2}p\left(1-p\right)}{d^{2}} \end{array}$$

where *z* = 1.96 corresponds to a 95% confidence level, *d* = 0.05 represents the permissible margin of error, and *P* = 0.5 reflects the expected proportion in the target population, *P* = 0.5 was used because the proportion of HR+ breast cancer patients with adequate osteoporosis-related knowledge in this setting was unknown; therefore, *P* = 0.5 was chosen to provide the most conservative (largest) sample size estimate. According to this calculation, the minimum sample size needed was 384 participants. Because SEM was also performed, we additionally assessed sample adequacy for SEM using a commonly applied rule-of-thumb of 10–20 participants per observed variable (indicator). The SEM included 31 observed indicators used to estimate the latent constructs, and the final analytic sample size was 491, yielding a case-to-indicator ratio of 15.8:1 (491/31), which falls within the recommended range.

### Statistical analyses

This study was descriptive and exploratory. The outcomes were the Knowledge, Attitudes, and Practices (KAP) dimension scores regarding osteoporosis (and their interrelationships), which together provide an overall profile of health literacy and self-management in HR+ breast cancer patients. Structural equation modeling (SEM) was used to explore the hypothesized relationships among these three dimensions, including the potential mediating role of attitudes between knowledge and practices. Statistical analysis was conducted using R 4.3.2 and Stata 18.0. The distribution of KAP dimension scores was empirically assessed for normality. The scores did not meet normality assumptions; therefore, continuous variables are presented as median (IQR). Group comparisons were performed using the Wilcoxon–Mann–Whitney test for two groups and the Kruskal–Wallis test for three or more groups. Correlations among dimension scores were evaluated using Spearman's rank correlation. Spearman's correlation was selected because the KAP scores did not meet normality assumptions. For regression analyses, KAP dimension scores were dichotomized using the median of each dimension as the cutoff, and univariate and multivariate logistic regression analyses were conducted to identify factors associated with KAP. Variables with *P* < 0.10 in univariate analyses were entered into multivariate models. A two-sided *P* value < 0.05 was considered statistically significant. Based on the KAP theoretical framework, SEM was used to examine the mediating role of attitudes between knowledge and practices. Model fit was evaluated using RMSEA, SRMR, TLI, CFI, and χ^2^/df, with RMSEA < 0.08, SRMR < 0.08, and TLI/CFI > 0.8 indicating acceptable fit. Correlated error terms were added between items with similar phrasing within the same dimension to improve model fit, following modification indices.

## Results

### Demographic characteristics and KAP scores

A total of 758 HR+ breast cancer patients were invited to participate in this study, and 610 completed the questionnaire (completion rate 80.5%). Data cleaning led to the exclusion of several cases: 8 for response times exceeding 1,800 s, 67 due to missing informed consent, 24 who were not HR+ breast cancer patients, and 20 due to logically contradictory selections within multiple-choice items (e.g., selecting the option “Uncertain” together with one or more specific answer options in the same item), which prevented unambiguous scoring according to the pre-specified questionnaire grading rules. This process resulted in 491 valid cases included in the final analysis (analytic inclusion rate 64.8%). Key demographic characteristics showed that 38.5% of participants had undergone more than 3 years of endocrine therapy, with the largest age group being 51–60 years (42.6%). Endocrine therapy medications were collected in the questionnaire (Item 1.1: Tamoxifen, anastrozole, letrozole, exemestane, or other). HR+ breast cancer patients had insufficient knowledge (mean 9.69, SD 5.93), moderate attitudes (mean 29.50, SD 4.55), and moderate practices (mean 35.85, SD 4.75) (Table 1).

**Table 1:** Baseline table.

***N* = 491**	***N* (%)**	**Knowledge**	***P***	**Attitudes**	***P***	**Practices**	***P***
		**median[IQR]**		**median[IQR]**		**median[IQR]**	
**Total score**	491 (100.0)	10.00 [4.00, 14.50]		30.00 [26.00, 32.00]		36.00 [32.50, 39.00]	
**Duration of endocrine therapy**							
Within 1 month	12(2.4)	10.00 [8.50, 13.25]	< 0.001	28.00 [23.50, 34.25]	0.085	34.50 [30.50, 38.25]	< 0.001
1-6 months	47(9.6)	6.00 [3.00, 11.00]		28.00 [24.50, 31.50]		34.00 [30.00, 36.50]	
6 months−1 year	99(20.2)	8.00 [3.00, 13.00]		30.00 [25.00, 32.00]		35.00 [31.00, 38.00]	
1 year−3 years	144(29.3)	9.00 [4.00, 14.25]		31.00 [27.75, 32.25]		36.00 [33.75, 39.00]	
Above 3 years	189(38.5)	12.00 [7.00, 15.00]		29.00 [26.00, 32.00]		37.00 [34.00, 40.00]	
**Age**							
Below 40 years old	48 (9.8)	11.00 [8.00, 16.00]	< 0.001	30.00 [26.75, 32.00]	0.028	37.00 [34.00, 39.25]	< 0.001
41–50 years old	90 (18.3)	10.00 [5.00, 15.00]		30.00 [25.00, 32.75]		36.00 [31.25, 38.75]	
51–60 years old	209 (42.6)	10.00 [4.00, 15.00]		29.00 [25.00, 32.00]		37.00 [34.00, 39.00]	
61–70 years old	104 (21.2)	10.00 [6.00, 14.00]		31.00 [28.00, 32.25]		37.00 [33.00, 40.00]	
Above 71 years old	40 (8.1)	4.00 [1.00, 9.00]		27.50 [25.00, 32.00]		33.00 [29.00, 35.25]	
**Marital status**							
Single	10 (2.0)	13.50 [5.75, 17.00]	0.416	31.00 [28.00, 37.50]	0.570	39.50 [35.00, 41.50]	0.262
Married	468 (95.3)	10.00 [4.00, 14.00]		30.00 [26.00, 32.00]		36.00 [32.00, 39.00]	
Divorced/widowed	13 (2.7)	12.00 [7.00, 15.00]		28.00 [26.00, 33.00]		36.00 [33.00, 39.00]	
**Education**							
Primary school and below	81 (16.5)	5.00 [2.00, 11.00]	< 0.001	30.00 [26.00, 32.00]	0.135	33.00 [31.00, 36.00]	< 0.001
Junior high school	188 (38.3)	9.00 [4.00, 13.00]		30.00 [25.00, 32.00]		36.00 [33.00, 38.00]	
High school/technical school	158 (32.2)	10.00 [5.00, 15.75]		29.00 [25.00, 32.00]		37.00 [32.25, 40.00]	
Bachelor's degree/college	64 (13.0)	14.00 [11.00, 18.00]		31.00 [27.00, 34.00]		38.00 [35.00, 41.00]	
**Type of occupation**							
Regular company employee	133 (27.1)	9.00 [3.00, 14.00]	< 0.001	29.00 [24.00, 32.00]	0.238	36.00 [31.00, 39.00]	0.004
Manual labor-intensive work	74 (15.1)	6.00 [3.00, 10.00]		30.00 [27.25, 32.75]		35.00 [33.00, 38.00]	
Homemaker	78 (15.9)	8.00 [4.00, 13.00]		30.00 [27.00, 32.00]		34.50 [31.00, 37.75]	
Retired	167 (34.0)	12.00 [7.00, 15.50]		29.00 [26.00, 32.00]		37.00 [34.00, 40.00]	
Other	39 (7.9)	14.00 [10.00, 16.00]		31.00 [26.00, 32.00]		36.00 [33.50, 39.00]	
**Have you experienced breast cancer metastasis**							
Yes	176(35.8)	9.00 [4.00, 14.00]	0.014	31.00 [28.00, 33.00]	< 0.001	37.00 [34.00, 39.00]	< 0.001
No	250(50.9)	11.00 [6.00, 15.00]		29.00 [25.00, 32.00]		37.00 [33.00, 40.00]	
Uncertain	65(13.2)	8.00 [2.00, 13.00]		27.00 [24.00, 32.00]		33.00 [30.00, 37.00]	
**Have you been diagnosed with osteoporosis**							
Yes	77(15.7)	13.00 [10.00, 16.00]	< 0.001	31.00 [28.00, 34.00]	0.013	38.00 [35.00, 40.00]	< 0.001
No	166(33.8)	10.50 [7.00, 15.00]		29.00 [26.00, 32.00]		37.00 [34.00, 40.00]	
Uncertain	248(50.5)	7.00 [2.75, 12.25]		30.00 [25.00, 32.00]		35.00 [31.00, 38.00]	
Demographic variables - multiple choice questions						Options			
**1.1 Currently, what medication are you receiving for endocrine therapy?**									
a. Tamoxifen					87 (17.7%)				
b. Anastrozole					79 (16.1%)				
c. Letrozole					172 (35%)				
d. Exemestane					160 (32.6%)				
e. Other					183 (37.3%)				
**6. Who usually takes care of you?**									
a. Spouse					410 (83.5%)				
b. Children					146 (29.7%)				
c. Parents					50 (10.2%)				
d. Other relatives					7 (1.4%)				
e. Live alone					12 (2.4%)				
f. Other (please specify)					2 (0.4%)				
**7. How do you pay for your medical expenses?**									
a. Urban and rural residents' medical insurance					184 (37.5%)				
b. Urban employee basic medical insurance					305 (62.1%)				
c. Other (please specify)					6 (1.2%)				

### Knowledge, attitudes and practices distribution

Responses to the knowledge dimension showed that 53.4% were unsure whether medications taken by HR+ breast cancer patients would cause osteoporosis (K3), and 67.6% were unsure about the relationship between osteoporosis and breast cancer (K5) and whether they would affect each other (K6). Meanwhile, 69.5% were unsure whether severe osteoporosis could lead to metastasis of breast cancer (K10), and 63.3% were unsure whether estrogen could be used to treat osteoporosis in HR+ breast cancer patients (K12).

In terms of related attitudes, 55.4% agreed that they should have a bone density test (A5), 54% agreed that it is more important to prevent osteoporosis caused by breast cancer than to treat it (A9), and 53% agreed that osteoporosis needs to be examined and treated in a hospital (A8). When it comes to whether or not they have developed osteoporosis (A1), 56.2% of the respondents' attitudes fluctuated. In addition, 13.2% did not think that osteoporosis would promote the development of breast cancer (A2) (Supplementary Table S2).

Responses to the practices items showed that only 30.1% were certain that they have had a bone density test (P 1). Results also showed that a combined 24.5% of participants were concerned about bumping or falling, leading them to refuse exercise (P5). On the other hand, 38.5% did not take the initiative to learn about the relevant knowledge related to their condition (Supplementary Table S3).

### Correlation analysis

In the correlation analysis (Spearman's rank correlation), knowledge was moderately and positively correlated with attitudes (*r* = 0.421, *P* < 0.001) and practices (*r* = 0.572, *P* < 0.001), and attitudes were moderately and positively correlated with practices (*r* = 0.483, *P* < 0.001) (Table 2). Given the cross-sectional design, these correlations should be interpreted as associations rather than evidence of directionality or causality.

**Table 2:** Spearman correlation analysis.

	**Knowledge**	**Attitudes**	**Practices**
Knowledge	1.000		
Attitudes	0.421 (*P* < 0.001)	1.000	
Practices	0.572 (*P* < 0.001)	0.483 (*P* < 0.001)	1.000

### Multivariate logistic regression analysis

Supplementary Table S4 shows the cut values. Multivariate logistic regression revealed that duration of endocrine therapy >3 years (OR = 0.22, 95% CI: 0.06–0.77, *P* = 0.018), undergraduate or college education (OR = 11.49, 95% CI: 3.75–35.16, *P* < 0.001), homemaker (OR = 2.16, 95% CI: 1.03–4.52, *P* = 0.041), retirement (OR = 4.11, 95% CI: 2.14–7.88, *P* < 0.001), other occupations (OR = 8.69, 95% CI: 3.23–23.40, *P* < 0.001), and self-reported undiagnosed or uncertain osteoporosis status (OR = 0.30, 95% CI: 0.15–0.60, *P* = 0.001; OR = 0.25, 95% CI: 0.13–0.48, *P* < 0.001) were independently associated with knowledge (Supplementary Table S5).

For attitudes, higher knowledge score (OR = 1.13, 95% CI: 1.09–1.17, *P* < 0.001), absence or uncertainty of breast cancer metastasis (OR = 0.41, 95% CI: 0.27–0.63, *P* < 0.001; OR = 0.31, 95% CI: 0.16–0.58, *P* < 0.001), and self-reported undiagnosed osteoporosis status (OR = 0.53, 95% CI: 0.30–0.96, *P* = 0.037) showed significant associations (Supplementary Table S6).

Regarding practices, higher knowledge (OR = 1.13, 95% CI: 1.08–1.19, *P* < 0.001) and attitudes scores (OR = 1.21, 95% CI: 1.14–1.29, *P* < 0.001), as well as higher education levels—junior high school (OR = 3.81, 95% CI: 1.75–8.33, *P* = 0.001), high school or technical school (OR = 5.10, 95% CI: 2.01–12.93, *P* = 0.001), and undergraduate or college (OR = 4.83, 95% CI: 1.47–15.93, *P* = 0.010)—were independently associated with proactive practices (Supplementary Table S7).

### Structural equation modeling

**Figure 1:**
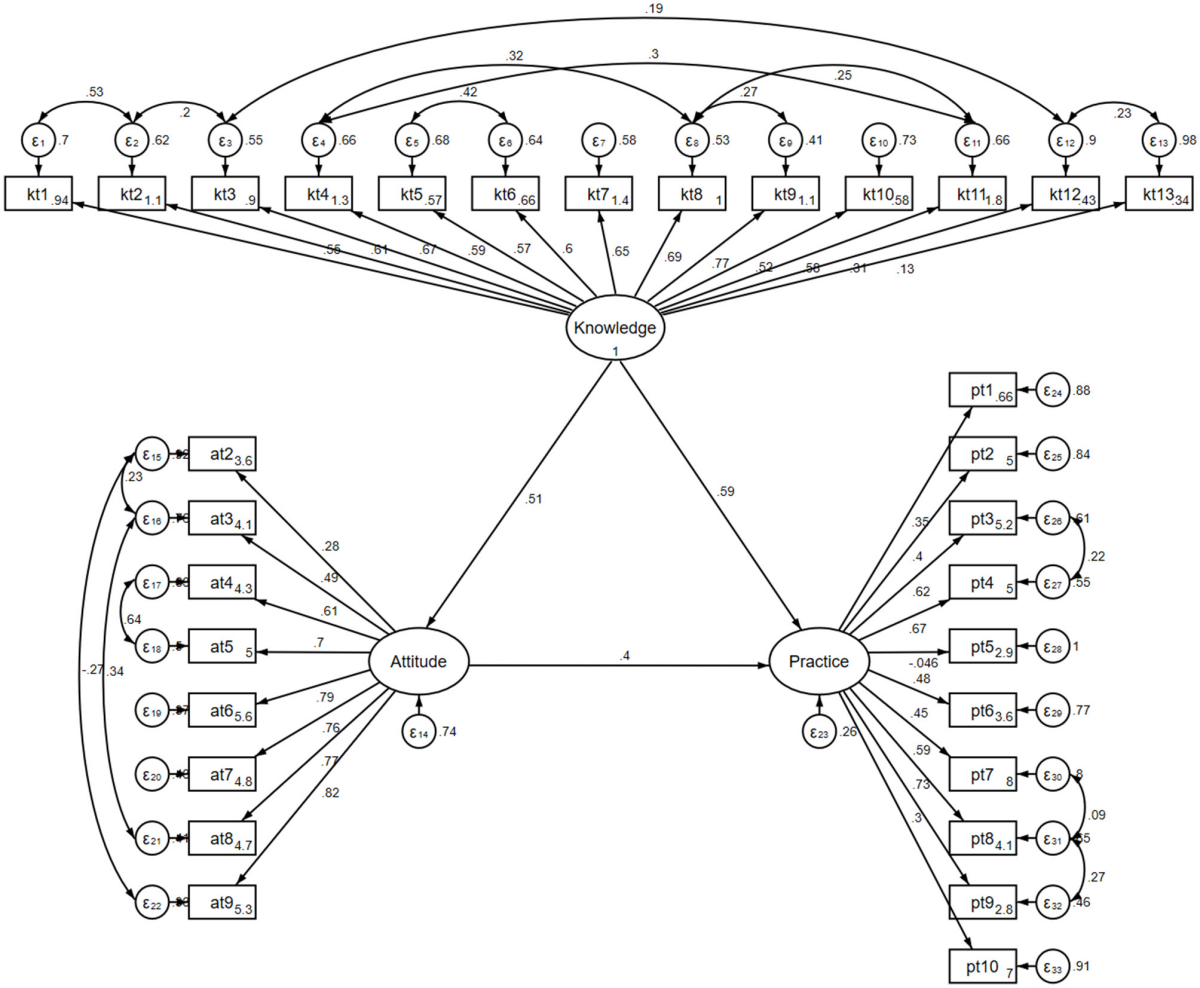
SEM model.

The outcomes of the analysis on direct and indirect effects revealed that knowledge exerted a direct influence on attitudes (β = 0.509, *P* < 0.001), while both knowledge (β = 0.585, *P* < 0.001) and attitudes (β = 0.402, *P* < 0.001) directly impacted practices. The SEM demonstrated acceptable fit (RMSEA = 0.074, SRMR = 0.079, TLI = 0.813, CFI = 0.833; Supplementary Table S8). Additionally, knowledge indirectly affected practices through its influence on attitudes (β = 0.205, *P* < 0.001) (Table 3, Figure 1).

**Table 3:** Analysis of direct and indirect effects.

**Model paths**		**Total effects**		**Direct effect**		**Indirect effect**	
		β **(95% CI)**	***P***	β **(95% CI)**	***P***	β **(95% CI)**	***P***
Total attitudes score	Total knowledge score	0.509 (0.429, 0.590)	< 0.001	0.509 (0.429, 0.590)	< 0.001		
Total practices score	Total attitudes score	0.402 (0.310, 0.494)	< 0.001	0.402 (0.310, 0.494)	< 0.001		
	Total knowledge score	0.790 (0.729, 0.851)	< 0.001	0585 (0.497, 0.674)	< 0.001	0.205 (0.152, 0.257)	< 0.001

## Discussion

HR+ breast cancer patients exhibit insufficient knowledge and moderate attitudes and practices concerning osteoporosis. Our findings suggest that targeted education may be particularly relevant for patients with lower educational attainment and for those with longer endocrine therapy duration, although the effectiveness of such approaches needs to be evaluated in future studies.

The study's findings regarding significant differences in knowledge, attitudes, and practices among HR+ breast cancer patients present valuable insights into factors influencing osteoporosis management. Participants with longer durations of endocrine therapy exhibited higher knowledge, attitudes, and practices scores concerning osteoporosis management. This pattern is in line with prior reports showing that longer treatment duration is associated with higher health-related knowledge and self-management behaviors in other clinical settings (14). These findings align with the recent study by Ma et al. (13), which similarly identified education level and treatment duration as significant factors influencing osteoporosis-related knowledge and practices. However, our study extends these findings by specifically focusing on HR+ breast cancer patients and demonstrating the mediating role of attitudes in the knowledge-practices relationship through structural equation modeling. The geographical and cultural context of our Nantong City cohort provides additional insights into how regional factors may influence patient behaviors and attitudes toward osteoporosis management.

Moreover, individuals with higher education levels demonstrated better knowledge, attitudes, and practices related to osteoporosis. Notably, patients aged >71 years showed substantially lower knowledge in the univariate analysis, which may relate to lower digital health literacy, age-related cognitive decline, or a greater focus on immediate cancer survivorship concerns rather than long-term bone health management. This aligns with existing literature highlighting the positive correlation between education level and health literacy across various populations (15–17). Multivariate logistic regression reinforces this association, indicating that individuals with a undergraduate or college education are significantly more likely to possess adequate knowledge. Additionally, a history of breast cancer metastasis was associated with lower knowledge and attitudes scores. Because our survey did not capture detailed clinical, psychosocial, or health-system factors, the specific reasons for this difference cannot be determined. One possible interpretation is that in metastatic care, immediate priorities related to cancer control and symptom management may reduce attention to long-term bone health education and self-management. Future studies incorporating qualitative assessments may help identify barriers specific to this subgroup. Furthermore, patients diagnosed with osteoporosis showed higher knowledge scores than those without or uncertain about an osteoporosis diagnosis. A plausible explanation is that a confirmed diagnosis often triggers physician-initiated counseling, and treatment discussions, which may improve osteoporosis-related knowledge and awareness. By contrast, patients without a diagnosis or those uncertain about their osteoporosis status may have had fewer opportunities for targeted communication and follow-up, which could contribute to lower knowledge. However, osteoporosis status in this study was self-reported and was not verified by medical record review or mandatory DXA assessment; therefore, these comparisons should be interpreted cautiously and should not be taken to indicate the presence or absence of osteoporosis, which is multifactorial. Osteoporosis status was self-reported rather than clinically adjudicated for all participants; therefore, misclassification of osteoporosis status is possible and should be considered when interpreting these comparisons.

The correlation analysis and SEM further elucidated the interplay between knowledge, attitudes, and practices. Significant positive correlations were observed between knowledge and attitudes, knowledge and practices, and attitudes and practices, indicating a cohesive relationship between these components. The SEM revealed that knowledge directly influenced attitudes and practices, with attitudes serving as a mediator between knowledge and practices. These findings are compatible with the KAP framework, in which knowledge and attitudes are related to health practices; however, the cross-sectional design does not allow inference about temporal order or causality (18, 19).

The distribution of responses in the knowledge dimension reveals varied levels of awareness and understanding among HR+ breast cancer patients regarding osteoporosis-related concepts. While a substantial proportion of participants correctly identified factors such as the association between menopause and osteoporosis, the increased risk of osteoporosis in breast cancer patients, and the potential impact of certain medications on bone health, there were still notable gaps in knowledge. For instance, a significant number of participants were uncertain about the relationship between osteoporosis and breast cancer or were unaware of symptoms indicative of osteoporosis. These findings suggest a need for targeted educational interventions to enhance awareness and knowledge regarding osteoporosis among HR+ breast cancer patients. Recommendations for improvement may include developing educational materials tailored to the specific information needs of this population, disseminating information through multiple channels such as healthcare providers, patient support groups, and social media platforms, and incorporating interactive elements to facilitate learning and retention of key concepts (20–22).

In the attitudes dimension, participants exhibited a range of perspectives regarding their beliefs and perceptions related to osteoporosis and its management. While many participants expressed concerns about the potential impact of osteoporosis on their daily lives and the progression of breast cancer, there were also notable areas of uncertainty or disagreement. For example, a considerable proportion of participants were unsure about the necessity of undergoing bone density testing, or believed that osteoporosis treatment required more expensive medical examination and hospital-based treatment. These findings underscore the importance of addressing misconceptions and uncertainties surrounding osteoporosis management among HR+ breast cancer patients. To address these gaps, interventions could focus on providing clear and accurate information about the importance of preventitive measures, promoting positive attitudes toward screening and treatment, and addressing fears or misconceptions that may act as barriers to proactive health behaviors (23–25).

In the practices dimension, participants demonstrated varying levels of engagement in behaviors aimed at preventing or managing osteoporosis. While a significant number of participants reported undergoing bone density testing and adhering to recommended treatments, there were also areas where practices could be improved. For instance, a notable proportion of participants reported uncertainty or reluctance regarding dietary adjustments, regular exercise, or calcium supplementation, which are key components of osteoporosis management. Additionally, concerns about the potential risks associated with exercise highlight the need for targeted interventions to address fears and misconceptions that may hinder engagement in physical activity. Practical recommendations may include providing tailored guidance on diet and exercise, offering resources or support for behavior change, and emphasizing the importance of a proactive approach to bone health within the context of breast cancer survivorship care (26–28). In our sample, only 30.1% of participants reported having undergone bone density testing. While physicians play a central role in initiating screening and counseling, shared decision-making also requires that patients recognize the value of these recommendations; insufficient knowledge or low perceived necessity may reduce follow-through even when testing is suggested.

This study had limitations. In our clinical setting, adjuvant bisphosphonates (e.g., zoledronic acid) are part of standard care for postmenopausal patients or those at high fracture risk. This routine clinical management and related counseling may increase bone health awareness and could have influenced the KAP scores observed. Firstly, the cross-sectional design of the study limits the ability to establish causality between variables, as it only captures a snapshot of the participants' KAP at a specific point in time. Secondly, the study was conducted at a single oncology hospital in Nantong City, potentially limiting the generalizability of the findings to broader populations of HR+ breast cancer patients. In addition, because only female patients were included, the findings may not be generalizable to male breast cancer patients. Furthermore, the use of convenience sampling and QR code-based data collection may have introduced selection bias, potentially favoring younger, more highly educated, or more tech-savvy participants who were able to complete a smartphone-based questionnaire, which may affect the representativeness of the sample. In addition, higher educational attainment is often associated with greater health literacy, which may introduce reporting bias because more educated participants may better understand and complete a structured questionnaire; therefore, their scores may partly reflect questionnaire comprehension rather than only true osteoporosis-related knowledge. Although these exclusions were based on prespecified, scoring-related criteria, we acknowledge that contradictory responses may be more common among participants with lower comprehension, which could slightly affect representativeness. Lastly, reliance on self-reported data through questionnaires may introduce recall bias and social desirability bias, impacting the accuracy and reliability of the reported KAP scores. In addition, osteoporosis diagnosis status was self-reported and not systematically confirmed by DXA or medical record review for all participants, which may have introduced misclassification. Additionally, the self-developed questionnaire, while showing good reliability (Cronbach's alpha = 0.841), may benefit from more extensive psychometric testing including construct validity and factor analysis in future studies. Cultural, social, and economic factors specific to the Nantong City context may limit the generalizability of findings to other populations with different backgrounds.

Based on our findings, healthcare providers should consider implementing targeted educational interventions in clinical settings. These interventions should be tailored according to patients‘ education levels, with simplified materials for those with lower educational attainment and more comprehensive resources for highly educated patients. For patients with longer treatment durations, educational programs should focus on maintaining engagement and updating knowledge about evolving treatment protocols. Clinical pathways should incorporate regular assessment of patients' knowledge, attitudes, and practices regarding osteoporosis, with personalized education plans developed based on individual assessment results. Additionally, healthcare teams should address specific misconceptions identified in our study, such as the relationship between osteoporosis and breast cancer progression, and provide clear guidance on safe exercise practices to overcome fears of physical activity.

In conclusion, HR+ breast cancer patients exhibit insufficient knowledge, positive attitudes, and suboptimal practices regarding osteoporosis. Given the significant associations identified between education levels and KAP scores, tailored educational interventions should be implemented targeting HR+ breast cancer patients, especially those with lower education levels, to improve their knowledge, attitudes, and practices concerning osteoporosis.

## Data Availability

The original contributions presented in the study are included in the article/Supplementary material, further inquiries can be directed to the corresponding authors.

## References

[1] Sung H Ferlay J Siegel RL Laversanne M Soerjomataram I Jemal A . Global cancer statistics 2020: GLOBOCAN estimates of incidence and mortality worldwide for 36 cancers in 185 countries. CA Cancer J Clin. (2021) 71:209–49. doi: 10.3322/caac.21660 33538338

[2] Fujioka T Katsuta L Kubota K Mori M Kikuchi Y Kato A . Classification of breast masses on ultrasound shear wave elastography using convolutional neural networks. Ultrason Imaging. (2020) 42:213–20. doi: 10.1177/0161734620932609 32501152

[3] Fisher LAB Ahmed O Chalchal HI Deobald R El-Gayed A Graham P . Outcomes of rural men with breast cancer: a multicenter population based retrospective cohort study. Cancers. (2023) 15:1995. doi: 10.3390/cancers15071995 37046656 PMC10093701

[4] Cardoso F Kyriakides S Ohno S Penault-Llorca F Poortmans P Rubio IT . Early breast cancer: ESMO clinical practice guidelines for diagnosis, treatment and follow-up†. Ann Oncol. (2019) 30:1194–220. doi: 10.1093/annonc/mdz173 31161190

[5] Howlader N Altekruse SF Li CI Chen VW Clarke CA Ries LA . US incidence of breast cancer subtypes defined by joint hormone receptor and HER2 status. J Natl Cancer Inst. (2014) 106:dju055. doi: 10.1093/jnci/dju055 24777111 PMC4580552

[6] An KC. Selective estrogen receptor modulators. Asian Spine J. (2016) 10:787–91. doi: 10.4184/asj.2016.10.4.787 27559463 PMC4995266

[7] Diana A Carlino F Giunta EF Franzese E Guerrera LP Di Lauro V . Cancer treatment-induced bone loss (CTIBL): state of the art and proper management in breast cancer patients on endocrine therapy. Curr Treat Options Oncol. (2021) 22:45. doi: 10.1007/s11864-021-00835-2 33864145 PMC8052225

[8] Edwards BJ Raisch DW Shankaran V McKoy JM Gradishar W Bunta AD . Cancer therapy associated bone loss: implications for hip fractures in mid-life women with breast cancer. Clin Cancer Res. (2011) 17:560–8. doi: 10.1158/1078-0432.CCR-10-1595 21288927 PMC3058419

[9] Hadji P. Cancer treatment-induced bone loss in women with breast cancer. Bonekey Rep. (2015) 4:692. doi: 10.1038/bonekey.2015.60 26029361 PMC4440228

[10] Aerts C Revilla M Duval L Paaijmans K Chandrabose J Cox H . Understanding the role of disease knowledge and risk perception in shaping preventive behavior for selected vector-borne diseases in Guyana. PLoS Negl Trop Dis. (2020) 14:e0008149. doi: 10.1371/journal.pntd.0008149 32251455 PMC7170267

[11] Liao L Feng H Jiao J Zhao Y Ning H. Nursing assistants' knowledge, attitudes and training needs regarding urinary incontinence in nursing homes: a mixed-methods study. BMC Geriatr. (2023) 23:39. doi: 10.1186/s12877-023-03762-z 36683023 PMC9867858

[12] Mumena WA. Maternal knowledge, attitude and practices toward free sugar and the associations with free sugar intake in children. Nutrients. (2021) 13:4403. doi: 10.3390/nu13124403 34959955 PMC8706702

[13] Ma Y Li Y Lu W Tang L Li P Qiu J . A cross-sectional study on knowledge, attitude and practice of osteoporosis prevention in patients with breast cancer undergoing endocrine therapy: a cross-sectional study. Asia Pac J Oncol Nurs. (2025) 12:100678. doi: 10.1016/j.apjon.2025.100678 40271527 PMC12017996

[14] Johnson AE Routh S Taylor CN Leopold M Beatty K McNamara DM . Developing and implementing an mHealth heart failure self-care program to reduce readmissions: randomized controlled trial. JMIR Cardio. (2022) 6:e33286. doi: 10.2196/33286 35311679 PMC8981015

[15] Mosli HH Kutbi HA Alhasan AH Mosli RH. Understanding the interrelationship between education, income, and obesity among adults in Saudi Arabia. Obes Facts. (2020) 13:77–85. doi: 10.1159/000505246 31955158 PMC7098293

[16] Echeverri M Anderson D Haas JM Johnson ME Serrano FSA Nápoles AM. Testing the preliminary validity of a multidimensional framework for studying the effects of cancer health literacy on cancer screening behaviors among diverse populations. Int J Environ Res Public Health. (2020) 17:2987. doi: 10.3390/ijerph17092987 32344860 PMC7246920

[17] Negesa LB Magarey J Rasmussen P Hendriks JML. Patients' knowledge on cardiovascular risk factors and associated lifestyle behaviour in Ethiopia in 2018: a cross-sectional study. PLoS ONE. (2020) 15:e0234198. doi: 10.1371/journal.pone.0234198 32497079 PMC7271995

[18] Muhoza P Saleem H Faye A Tine R Diaw A Kante AM . Behavioral determinants of routine health information system data use in Senegal: a qualitative inquiry based on the integrated behavioral model. Glob Health Sci Pract. (2022) 10:e2100686. doi: 10.9745/GHSP-D-21-00686 36332064 PMC9242607

[19] Moradi A Soltani R Shamsi M Moradzadeh R. Effects of online social media on improving mothers' behaviors towards preventing their children's otitis media based on the PRECED model: a randomized educational intervention trial. BMC Pediatr. (2023) 23:216. doi: 10.1186/s12887-023-04016-y 37147633 PMC10161150

[20] Bennett MJ Center JR Perry L. Establishing consensus recommendations for long-term osteoporosis care for patients who have attended an Australian fracture liaison service: a Delphi study. Osteoporos Int. (2024) 35:373–89. doi: 10.1007/s00198-024-07014-7 38267665 PMC10867051

[21] Rubæk M Broholm-Jørgensen M Andersen S Jakobsen PR Rothmann MJ Langdahl B . Development of a program theory for osteoporosis patient education in Denmark: a qualitative study based on realist evaluation. BMC Geriatr. (2024) 24:346. doi: 10.1186/s12877-024-04957-8 38627654 PMC11022455

[22] Zeng YH Hao DJ. [Exploration and prospect of the whole course management model of osteoporosis]. Zhonghua Yi Xue Za Zhi. (2023) 103:2737–42. doi: 10.3760/cma.j.cn112137-20230413-00595 37723047

[23] Alani Q Yassir M Mansoor R Flayyih R Ali Saifuddin Yaqoob N Rafeeq H . Knowledge, attitude, and practices towards osteoporosis among adults in the United Arab Emirates (UAE) in 2023. Cureus. (2024) 16:e56084. doi: 10.7759/cureus.56084 38618442 PMC11011242

[24] Lyu FF Ramoo V Chui PL Ng CG. Perceptions toward exercise or mindful exercise participation among patients with primary osteoporosis: a qualitative study. Clin Nurs Res. (2024) 33:40–50. doi: 10.1177/10547738231198561 37970808

[25] Xu P Zhao N Wang J. Knowledge, attitude, and practice toward osteoporosis among patients with chronic kidney disease in Zhejiang. Medicine. (2024) 103:e38153. doi: 10.1097/MD.0000000000038153 38758880 PMC11098230

[26] Cheng WP Sun L Shen D Xu GH Jiang JW Gu HY. Effectiveness of mobile health platform-based continuity of care in osteoporosis prevention and treatment. Altern Ther Health Med. (2024) 30:144–9. 38466062

[27] Di Martino G Della Valle C Centorbi M Buonsenso A Fiorilli G Calcagno G . Enhancing behavioural changes: a narrative review on the effectiveness of a multifactorial APP-based intervention integrating physical activity. Int J Environ Res Public Health. (2024) 21:233. doi: 10.3390/ijerph21020233 38397722 PMC10888703

[28] Patel D Gorrell C Norris J Liu J. A narrative review of the pharmaceutical management of osteoporosis. Ann Jt. (2023) 8:25. doi: 10.21037/aoj-23-2 38529240 PMC10929303

