# Supplementary material for: Knowledge, attitudes, and practices regarding osteoporosis among hormone receptor-positive breast cancer patients: a cross-sectional study
Source: Front Public Health. 2026 Mar 5;14:1741278. doi: 10.3389/fpubh.2026.1741278 (PMC12999964; doi:10.3389/fpubh.2026.1741278)
Supplement: Supplementary file 1 [file Data_Sheet_1.docx]

**Table S1. Distribution of knowledge dimension responses**

| **Knowledge** |  |  |  |  |  |
| --- | --- | --- | --- | --- | --- |
|  | Yes | No | Uncertain |  |  |
| **1.** **Does menopause increase the risk of osteoporosis?** | 230(46.8%) | 8(1.6%) | 253(51.5%) |  |  |
| **2.** **Are breast cancer patients more prone to osteoporosis compared to the general population?** | 276(56.2%) | 8(1.6%) | 207(42.2%) |  |  |
| **3.** **Can medications taken by hormone receptor-positive breast cancer patients lead to osteoporosis?** | 220(44.8%) | 9(1.8%) | 262(53.4%) |  |  |
| **4.** **Which of the following symptoms are indicative of osteoporosis? (Multiple choices allowed)** | Yes |  |  | No |  |
| a. Fractures | 306(62.3%) |  |  | 185(37.7%) |  |
| b. Back pain | 258(52.5%) |  |  | 233(47.5%) |  |
| c. Chest tightness and palpitations | 86(17.5%) |  |  | 405(82.5%) |  |
| d. Kyphosis (hunchback) | 137(27.9%) |  |  | 354(72.1%) |  |
| e. Uncertain | 120(24.4%) |  |  | 371(75.6%) |  |
|  | Yes | No | Uncertain |  |  |
| **5.** **Are you aware of the relationship between osteoporosis and breast cancer?** | 122(24.8%) | 37(7.5%) | 332(67.6%) |  |  |
| **6.** **Do breast cancer and osteoporosis mutually influence each other?** | 148(30.1%) | 12(2.4%) | 331(67.4%) |  |  |
| **7.** **Which treatment methods are included in the management of hormone receptor-positive breast cancer patients? (Multiple choices allowed)** | Yes |  |  | No |  |
| a. Chemotherapy | 314(64%) |  |  | 177(36%) |  |
| b. Endocrine therapy | 343(69.9%) |  |  | 148(30.1%) |  |
| c. Radiation therapy | 193(39.3%) |  |  | 298(60.7%) |  |
| d. Surgery | 185(37.7%) |  |  | 306(62.3%) |  |
| e. Uncertain | 128(26.1%) |  |  | 363(73.9%) |  |
| **8.** **Are you aware of medications that may be associated with osteoporosis? (Multiple choices allowed)** | Yes |  |  | No |  |
| a. Endocrine therapy medications | 306(62.3%) |  |  | 185(37.7%) |  |
| b. Anticancer medications | 198(40.3%) |  |  | 293(59.7%) |  |
| c. Thyroid hormone medications | 60(12.2%) |  |  | 431(87.8%) |  |
| d. Overuse of vitamin D and calcium supplements | 60(12.2%) |  |  | 431(87.8%) |  |
| e. Uncertain | 188(38.3%) |  |  | 303(61.7%) |  |
|  | Promotes | Opposes | Can treat | Uncertain |  |
| **9.** **What is the relationship between endocrine therapy medications and osteoporosis?** | 276(56.2%) | 2(0.4%) | 3(0.6%) | 210(42.8%) |  |
|  | Yes | No | Uncertain |  |  |
| **10.** **Can severe osteoporosis lead to breast cancer metastasis?** | 123(25.1%) | 27(5.5%) | 341(69.5%) |  |  |
| **11.** **Are you familiar with medications that can alleviate or prevent osteoporosis? (Multiple choices allowed)** | Yes |  |  | No |  |
| a. Vitamin D | 280(57%) |  |  | 211(43%) |  |
| b. Calcium supplements | 423(86.2%) |  |  | 68(13.8%) |  |
| c. Calcitonin | 17(3.5%) |  |  | 474(96.5%) |  |
| d. Bisphosphonate drugs | 60(12.2%) |  |  | 431(87.8%) |  |
| e. Growth hormone drugs | 12(2.4%) |  |  | 479(97.6%) |  |
| f. Uncertain | 59(12%) |  |  | 432(88%) |  |
|  | Yes | No | Uncertain |  |  |
| **12.** **Can hormone receptor-positive breast cancer patients use estrogen therapy to treat osteoporosis?** | 104(21.2%) | 76(15.5%) | 311(63.3%) |  |  |
| **13.** **Is it effective to consume calcium supplements and bone broth to treat osteoporosis?** | 336(68.4%) | 50(10.2%) | 105(21.4%) |  |  |

**Table S2. Distribution of attitudes dimension responses**

| **Attitudes** | Strongly agree | Agree | Neutral | Disagree | Strongly disagree |
| --- | --- | --- | --- | --- | --- |
| **1.** **Do you believe that you have developed osteoporosis?** | 57(11.6%) | 119(24.2%) | 276(56.2%) | 10(2%) | 29(5.9%) |
| **2.** **Do you believe that osteoporosis can promote the progression of breast cancer?** | 41(8.4%) | 138(28.1%) | 247(50.3%) | 65(13.2%) | 0 (0%) |
| **3.** **Are you concerned that bone pain may be a sign of breast cancer bone metastasis or deterioration?** | 143(29.1%) | 200(40.7%) | 117(23.8%) | 26(5.3%) | 5(1%) |
| **4.** **Are you worried that osteoporosis will affect your daily life?** | 143(29.1%) | 228(46.4%) | 103(21%) | 16(3.3%) | 1(0.2%) |
| **5.** **Do you believe that you should undergo bone density testing?** | 73(14.9%) | 272(55.4%) | 137(27.9%) | 9(1.8%) | 0 (0%) |
| **6.** **Do you believe that breast cancer treatment will exacerbate osteoporosis?** | 66(13.4%) | 224(45.6%) | 180(36.7%) | 20(4.1%) | 1(0.2%) |
| **7.** **Do you believe that taking measures to prevent osteoporosis can prevent further development of breast cancer?** | 62(12.6%) | 201(40.9%) | 202(41.1%) | 26(5.3%) | 0 (0%) |
| **8.** **Do you believe that osteoporosis requires medical examination and treatment at the hospital?** | 73(14.9%) | 260(53%) | 145(29.5%) | 13(2.6%) | 0 (0%) |
| **9. Do you believe that prevention of osteoporosis caused by breast cancer is more important than treatment?** | 79(16.1%) | 265(54%) | 135(27.5%) | 12(2.4%) | 0 (0%) |

**Table S3. Distribution of practice dimension responses**

| **Practices** |  |  |  |  |  |
| --- | --- | --- | --- | --- | --- |
|  | Yes | No | Uncertain |  |  |
| **1.** **Have you ever had a bone density test?** | 148(30.1%) | 144(29.3%) | 199(40.5%) |  |  |
|  | Strongly agree | Agree | Neutral | Disagree | Strongly disagree |
| **2.** **If diagnosed with osteoporosis, do you undergo osteoporosis treatment?** | 277(56.4%) | 132(26.9%) | 70(14.3%) | 4(0.8%) | 8(1.6%) |
| **3.** **Do you adjust your diet, such as eating foods high in calcium, to prevent or improve osteoporosis?** | 173(35.2%) | 228(46.4%) | 72(14.7%) | 18(3.7%) | 0 (0%) |
| **4.** **Do you engage in regular exercise, such as walking or aerobics, to prevent or improve osteoporosis?** | 192(39.1%) | 178(36.3%) | 112(22.8%) | 9(1.8%) | 0 (0%) |
| **5.** **Do you refuse to exercise due to fear of bumps or falls?** | 50(10.2%) | 70(14.3%) | 101(20.6%) | 233(47.5%) | 37(7.5%) |
| **6.** **Do you regularly take calcium supplements in your daily life?** | 90(18.3%) | 146(29.7%) | 195(39.7%) | 47(9.6%) | 13(2.6%) |
| **7.** **Do you actively expose yourself to sunlight?** | 299(60.9%) | 182(37.1%) | 6(1.2%) | 3(0.6%) | 1(0.2%) |
| **8.** **Do you pay attention to symptoms such as lower back pain, fatigue, etc., in your daily life?** | 102(20.8%) | 208(42.4%) | 130(26.5%) | 51(10.4%) | 0 (0%) |
| **9.** **Do you actively seek knowledge about hormone receptor-positive breast cancer and osteoporosis?** | 82(16.7%) | 111(22.6%) | 104(21.2%) | 189(38.5%) | 5(1%) |
| **10.** **Are your lifestyle habits and routines regular?** | 367(74.7%) | 98(20%) | 12(2.4%) | 14(2.9%) | 0 (0%) |

**Table S4. Score distribution**

| **Cutoff Value: Median for Knowledge, Attitudes, and Practices** | N (%) |
| --- | --- |
| Knowledge dimension total score |  |
| **Total knowledge score >=10** | 255(51.9%) |
| **Total knowledge score <=9** | 236(48.1%) |
| Attitudes dimension total score |  |
| **Total attitudes score >=30** | 255(51.9%) |
| **Total attitudes score <=29** | 236(48.1%) |
| Practices dimension total scores |  |
| **Total practices score >=36** | 275(56.0%) |
| **Total practices score <=35** | 216(44.0%) |

**Table S5. Univariate and multivariate analysis for knowledge dimension**

| **Knowledge** | Univariate analysis |  | Multivariate analysis |  |
| --- | --- | --- | --- | --- |
|  | OR(95%CI) | P | OR(95%CI) | P |
| **Duration of endocrine therapy** |  |  |  |  |
| Within 1 month |  |  |  |  |
| 1-6 months | 0.47 (0.13,1.73) | 0.249 | 0.96 (0.40,2.31) | 0.932 |
| 6 months -1 year | 0.74 (0.22,2.51) | 0.618 | 0.86 (0.35,2.09) | 0.732 |
| 1 year -3 years | 0.95 (0.28,3.16) | 0.926 | 0.66 (0.24,1.82) | 0.426 |
| Above 3 years | 1.82 (0.55,6.03) | 0.315 | 0.22 (0.06,0.77) | 0.018 |
| **Age** |  |  |  |  |
| Below 40 years old |  |  |  |  |
| 41~50 years old | 0.50 (0.23,1.03) | 0.063 |  |  |
| 51~60 years old | 0.51 (0.26,0.99) | 0.051 |  |  |
| 61-70 years old | 0.49 (0.23,1.00) | 0.053 |  |  |
| Above 71 years old | 0.15 (0.06,0.38) | <0.001 |  |  |
| **Marital status** |  |  |  |  |
| Single |  |  |  |  |
| Married | 0.45 (0.10,1.64) | 0.253 |  |  |
| Divorced /widowed | 0.69 (0.11,3.90) | 0.673 |  |  |
| **Education** |  |  |  |  |
| Primary school and below |  |  |  |  |
| Junior high school | 2.05 (1.18,3.62) | 0.012 | 1.11 (0.56,2.19) | 0.767 |
| High school/technical school | 3.06 (1.75,5.50) | <0.001 | 1.89 (0.86,4.19) | 0.115 |
| Bachelor’s degree/ college | 14.51 (6.45,35.83) | <0.001 | 11.49 (3.75,35.16) | <0.001 |
| **Type of occupation** |  |  |  |  |
| Regular company employee |  |  |  |  |
| Manual labor-intensive work | 0.40 (0.21,0.73) | 0.003 | 1.37 (0.61,3.07) | 0.441 |
| Homemaker | 0.88 (0.50,1.54) | 0.648 | 2.16 (1.03,4.52) | 0.041 |
| Retired | 1.87 (1.18,2.99) | 0.008 | 4.11 (2.14,7.88) | <0.001 |
| Other | 3.59 (1.64,8.57) | 0.002 | 8.69 (3.23,23.40) | <0.001 |
| **Have you experienced breast cancer metastasis** |  |  |  |  |
| Yes |  |  |  |  |
| No | 1.50 (1.02,2.21) | 0.041 | 1.09 (0.69,1.72) | 0.708 |
| Uncertain | 0.90 (0.51,1.60) | 0.725 | 0.87 (0.45,1.71) | 0.694 |
| **Have you been diagnosed with osteoporosis** |  |  |  |  |
| Yes |  |  |  |  |
| No | 0.40 (0.21,0.72) | 0.003 | 0.30 (0.15,0.60) | 0.001 |
| Uncertain | 0.21 (0.12,0.38) | <0.001 | 0.25 (0.13,0.48) | <0.001 |

**Table S6. Univariate and multivariate analysis for attitudes dimension**

| **Attitudes** | Univariate analysis |  | Multivariate analysis |  |
| --- | --- | --- | --- | --- |
|  | OR(95%CI) | P | OR(95%CI) | P |
| **Knowledge** | 1.10 (1.07,1.14) | <0.001 | 1.13 (1.09,1.17) | <0.001 |
| **Duration of endocrine therapy** |  |  |  |  |
| Within 1 month |  |  |  |  |
| 1-6 months | 0.79 (0.22,3.04) | 0.726 |  |  |
| 6 months -1 year | 1.49 (0.44,5.33) | 0.521 |  |  |
| 1 year -3 years | 2.20 (0.67,7.75) | 0.196 |  |  |
| Above 3 years | 1.39 (0.43,4.83) | 0.589 |  |  |
| **Age** |  |  |  |  |
| Below 40 years old |  |  |  |  |
| 41~50 years old | 0.72 (0.35,1.45) | 0.357 |  |  |
| 51~60 years old | 0.59 (0.31,1.11) | 0.105 |  |  |
| 61-70 years old | 0.97 (0.48,1.94) | 0.925 |  |  |
| Above 71 years old | 0.54 (0.23,1.25) | 0.151 |  |  |
| **Marital status** |  |  |  |  |
| Single |  |  |  |  |
| Married | 0.73 (0.18,2.57) | 0.624 |  |  |
| Divorced /widowed | 0.42 (0.07,2.20) | 0.309 |  |  |
| **Education** |  |  |  |  |
| Primary school and below |  |  |  |  |
| Junior high school | 0.98 (0.58,1.66) | 0.949 |  |  |
| High school/technical school | 0.76 (0.44,1.30) | 0.314 |  |  |
| Bachelor’s degree/ college | 1.47 (0.76,2.89) | 0.256 |  |  |
| **Type of occupation** |  |  |  |  |
| Regular company employee |  |  |  |  |
| Manual labor-intensive work | 1.62 (0.91,2.91) | 0.100 |  |  |
| Homemaker | 1.29 (0.73,2.26) | 0.381 |  |  |
| Retired | 0.99 (0.62,1.56) | 0.949 |  |  |
| Other | 1.22 (0.60,2.52) | 0.585 |  |  |
| **Have you experienced breast cancer metastasis** |  |  |  |  |
| Yes |  |  |  |  |
| No | 0.46 (0.31,0.69) | <0.001 | 0.41 (0.27,0.63) | <0.001 |
| Uncertain | 0.36 (0.20,0.65) | 0.001 | 0.31 (0.16,0.58) | <0.001 |
| **Have you been diagnosed with osteoporosis** |  |  |  |  |
| Yes |  |  |  |  |
| No | 0.46 (0.26,0.80) | 0.006 | 0.53 (0.30,0.96) | 0.037 |
| Uncertain | 0.65 (0.38,1.09) | 0.109 | 1.16 (0.64,2.09) | 0.623 |

**Table S7. Univariate and multivariate analysis for practices dimension**

| **Practices** | Univariate analysis |  | Multivariate analysis |  |
| --- | --- | --- | --- | --- |
|  | OR(95%CI) | P | OR(95%CI) | P |
| **Attitudes** | 1.22 (1.17,1.27) | <0.001 | 1.13 (1.08,1.19) | <0.001 |
| **Knowledge** | 1.24 (1.18,1.31) | <0.001 | 1.21 (1.14,1.29) | <0.001 |
| **Duration of endocrine therapy** |  |  |  |  |
| Within 1 month |  |  |  |  |
| 1-6 months | 0.66 (0.18,2.53) | 0.526 | 1.64 (0.29,9.15) | 0.571 |
| 6 months -1 year | 1.27 (0.38,4.53) | 0.704 | 2.63 (0.51,13.62) | 0.251 |
| 1 year -3 years | 1.80 (0.55,6.33) | 0.335 | 3.18 (0.63,16.10) | 0.162 |
| Above 3 years | 2.87 (0.88,10.03) | 0.082 | 4.92 (0.99,24.44) | 0.051 |
| **Age** |  |  |  |  |
| Below 40 years old |  |  |  |  |
| 41~50 years old | 0.60 (0.29,1.22) | 0.165 | 0.72 (0.28,1.87) | 0.504 |
| 51~60 years old | 0.80 (0.41,1.52) | 0.503 | 1.41 (0.53,3.73) | 0.488 |
| 61-70 years old | 0.84 (0.41,1.70) | 0.637 | 1.50 (0.50,4.49) | 0.466 |
| Above 71 years old | 0.18 (0.07,0.45) | <0.001 | 0.69 (0.18,2.76) | 0.605 |
| **Marital status** |  |  |  |  |
| Single |  |  |  |  |
| Married | 0.54 (0.12,1.97) | 0.377 |  |  |
| Divorced /widowed | 0.50 (0.08,2.75) | 0.434 |  |  |
| **Education** |  |  |  |  |
| Primary school and below |  |  |  |  |
| Junior high school | 3.54 (2.03,6.36) | <0.001 | 3.81 (1.75,8.33) | 0.001 |
| High school/technical school | 4.50 (2.54,8.22) | <0.001 | 5.10 (2.01,12.93) | 0.001 |
| Bachelor’s degree/ college | 7.41 (3.61,15.92) | <0.001 | 4.83 (1.47,15.93) | 0.010 |
| **Type of occupation** |  |  |  |  |
| Regular company employee |  |  |  |  |
| Manual labor-intensive work | 0.76 (0.43,1.34) | 0.346 | 1.16 (0.48,2.80) | 0.742 |
| Homemaker | 0.69 (0.39,1.21) | 0.195 | 0.80 (0.34,1.85) | 0.597 |
| Retired | 1.55 (0.97,2.48) | 0.065 | 1.04 (0.50,2.16) | 0.911 |
| Other | 1.51 (0.73,3.23) | 0.271 | 1.04 (0.39,2.79) | 0.941 |
| **Have you experienced breast cancer metastasis** |  |  |  |  |
| Yes |  |  |  |  |
| No | 0.79 (0.53,1.17) | 0.239 | 0.61 (0.36,1.04) | 0.068 |
| Uncertain | 0.33 (0.18,0.59) | <0.001 | 0.47 (0.22,1.04) | 0.062 |
| **Have you been diagnosed with osteoporosis** |  |  |  |  |
| Yes |  |  |  |  |
| No | 0.60 (0.33,1.09) | 0.099 | 0.89 (0.42,1.88) | 0.754 |
| Uncertain | 0.29 (0.16,0.51) | <0.001 | 0.53 (0.25,1.11) | 0.092 |

**Table S8. Model fit indices for the structural equation model (SEM)**

| Indicators | Reference | Results |
| --- | --- | --- |
| RMSEA | <0.08Good | 0.074 |
| SRMR | <0.08Good | 0.079 |
| TLI | >0.8Good | 0.813 |
| CFI | >0.8Good | 0.833 |
